# Glucose regulation and physical performance among older people: the Helsinki Birth Cohort Study

**DOI:** 10.1007/s00592-018-1192-1

**Published:** 2018-07-21

**Authors:** Max J. Åström, Mikaela B. von Bonsdorff, Mia M. Perälä, Minna K. Salonen, Taina Rantanen, Eero Kajantie, Mika Simonen, Pertti Pohjolainen, Clive Osmond, Johan G. Eriksson

**Affiliations:** 1 Department of General Practice and Primary Health Care, University of Helsinki and Helsinki University Hospital, PO Box 20, 00014 Helsinki, Finland; 2 Folkhälsan Research Center, Helsinki, Finland; 3 Faculty of Sport and Health Sciences, Gerontology Research Center, University of Jyväskylä, Jyväskylä, Finland; 4 Public Health Promotion Unit, Department of Public Health Solutions, National Institute for Health and Welfare, Helsinki, Finland; 5 Hospital for Children and Adolescents, Helsinki University Central Hospital and University of Helsinki, Helsinki, Finland; 6 PEDEGO Research Unit, MRC Oulu, Oulu University Hospital and University of Oulu, Oulu, Finland; 7 Finnish Centre of Excellence in Intersubjectivity and Interaction, University of Helsinki, Helsinki, Finland; 8 Age Institute, Helsinki, Finland; 9 MRC Lifecourse Epidemiology Unit, University of Southampton, Southampton, UK

**Keywords:** Ageing, Diabetes mellitus, Glucose intolerance, Glucose tolerance test, Physical fitness, Epidemiology

## Abstract

**Aims:**

To assess whether disturbances in glucose regulation are associated with impairment in physical performance during a 10-year follow-up.

**Methods:**

475 Men and 603 women from the Helsinki Birth Cohort Study were studied. Glucose regulation was evaluated with a 2-h 75-g oral glucose tolerance test (OGTT) in 2001–2004. Subjects were categorised as having either impaired fasting glucose (IFG), impaired glucose tolerance (IGT), newly diagnosed diabetes or previously known diabetes. Physical performance was assessed approximately 10 years later using the validated senior fitness test (SFT). The relationship between glucose regulation and the overall SFT score was estimated using multiple linear regression models.

**Results:**

The mean age was 70.8 years for men and 71.0 years for women when physical performance was assessed. The mean SFT score for the whole population was 45.0 (SD 17.5) points. The SFT score decreased gradually with increased impairment in glucose regulation. Individuals with previously known diabetes had the lowest overall SFT score in the fully adjusted model (mean difference compared to normoglycaemic individuals − 11.56 points, 95% CI − 16.15 to − 6.98, *p* < 0.001). Both individuals with newly diagnosed diabetes and individuals with IGT had significantly poorer physical performance compared to those with normoglycaemia. No significant difference in physical performance was found between those with IFG and those with normoglycaemia.

**Conclusions:**

Among older people, impaired glucose regulation is strongly related with poor physical performance. More severe disturbances in glucose regulation are associated with a greater decrease in physical function, indicating the importance of diagnosing these disturbances at an early stage.

## Introduction

The global burden of diabetes among older people is growing. The age-standardised prevalence of diabetes has doubled since 1980 [1] and in the year 2017 globally over 120 million people aged 65 years or older had diabetes [2]. In Finland in 2005, approximately 20% of adults over 65 years were diagnosed with diabetes, whereas 30% of the older population had prediabetes, consisting of impaired glucose tolerance (IGT) or impaired fasting glucose (IFG) or a combination of the two [3]. The prevalence of diabetes and prediabetes in Finland is similar to the prevalence in other European countries [2]. Prediabetes has an annual risk of 5–10% of progressing to diabetes [4] and it also increases the risk for microvascular complications traditionally associated with diabetes [5, 6]. As the population ages due to increased life expectancy and a fall in fertility, non-communicable diseases such as heart disease and diabetes will become even more important causes of morbidity and mortality, regardless of income level [7]. This calls for effective primary and secondary interventions in older people to reduce the disease burden and health care costs [8].

Decreased physical performance, an important predictor of disability and functional decline, has been shown to have negative consequences on the daily life of older people [9–11]. Diabetes is one of the major causes of physical limitation and individuals with diabetes have approximately 50–80% greater risk of disability compared to those without diabetes [12]. Age-related sarcopenia, characterised by loss of muscle strength and muscle quality, is accelerated by diabetes especially in the lower extremities [13, 14]. This increases the prevalence of mobility limitation and frailty at an earlier age, which reduces quality of life and results in loss of independence and institutionalisation [15, 16]. Disability and poor physical performance also lead to a rise in health care costs and an increased mortality rate [17, 18].

Although there is an apparent correlation between diabetes and physical disability, most studies focusing on this subject have been cross-sectional [12, 19, 20]. On the other hand, longitudinal studies have often relied on self-reported diagnosis of diabetes or self-reported physical performance, mainly focusing on activities of daily living (ADL), instrumental activities of daily living (IADL) and motility [21–23]. Furthermore, there are no longitudinal studies assessing the relationship between prediabetes and physical performance.

To address this question, we studied the association between glucose regulation at an average age of 61 years and the objectively measured physical performance evaluated with the Senior Fitness Test (SFT) approximately 10 years later using data from the Helsinki Birth Cohort Study.

## Materials and methods

### Study population

The Helsinki Birth Cohort Study (HBCS) includes 13,345 individuals born between 1934 and 1944 at the Helsinki University Central Hospital or the Helsinki City Maternity Hospital [24]. All subjects included in this study cohort attended child welfare clinics in Helsinki and were still living in Finland in 1971, when all Finnish residents received a personal identification number [25]. A random sample of 2902 from those 8760 individuals who were born at the Helsinki University Central Hospital was invited to a baseline clinical examination in the year 2000. A total of 2003 cohort members participated in an examination conducted between 2001 and 2004. In 2011, members of the clinical cohort still alive and living within a 100-km radius of Helsinki were invited to participate in a clinical follow-up examination. A total of 1094 subjects participated in this clinical examination between 2011 and 2013. The cohort members that participated only in the baseline examination (*n* = 925) were older, more frequently men and smokers and they had higher BMI (all *p* < 0.028) compared to those who were included in our study. The main reasons for declining invitation to the follow-up examination were related to personal or a family member’s health conditions. This study includes the 1078 subjects who had adequate information on physical performance and glucose regulation [26]. The study was approved by the Ethics Committee of Epidemiology and Public Health of the Hospital District of Helsinki and Uusimaa and that of the National Public Health Institute, Helsinki and follows the guidelines of the Declaration of Helsinki. All participants gave a written, informed consent.

### Glucose regulation

Fasting plasma glucose was measured in all subjects at the baseline clinical examination in 2001 to 2004. A standard 2-h 75-g oral glucose tolerance test (OGTT) was conducted in all individuals, except for those with previously known type 1 or type 2 diabetes (*n* = 50), defined by self-report, medical records or use of medication for diabetes. The World Health Organization 1999 criteria [27] were used for diagnosing diabetes, IGT, and IFG. Subjects reporting a diagnosis of diabetes or taking medications for diabetes before the clinical examination were classified as having previously known diabetes. Those diagnosed with type 2 diabetes for the first time at the OGTT were classified as having newly diagnosed diabetes. Subjects meeting the criteria for both IGT and IFG were categorised as having IGT.

### Physical performance

Subjects in the clinical follow-up were assessed for physical performance with the validated Senior Fitness Test (SFT) [28, 29]. A modified test battery consisting of five tests was carried out: (1) 30-s chair stand: number of full stands completed in 30 s with arms folded across chest to assess lower body strength, (2) Arm curl: number of bicep curls recorded in 30 s holding hand weight (3 kg for men and 2 kg for women) to assess upper body strength, (3) Back scratch: with one hand reaching over shoulder and the other one up middle back, number of centimetres between extended middle fingers to assess upper body (shoulder) flexibility, (4) Chair sit-and-reach: sitting at the front of chair with leg extended, number of centimetres between extended fingers and tip of toe to assess lower body (hamstring) flexibility, (5) 6 min walk: number of meters walked in 6 min to assess aerobic endurance. Measurements were performed by trained research assistants [26]. Subjects were rated for each test using percentile tables of normative data for 5-year age groups [29]. The rating varied between 1 and 20, based on 5-‰ ranges, with 1 indicating a test result below the fifth percentile and 20 indicating a test result in the top 5 ‰. Finally, the overall score was calculated as the sum of the normalised ratings for all five SFT items. The overall score varied between 5 and 100.

### Covariates

Questionnaires were used at the baseline clinical examination to assess current health situation, use of medication, educational attainment and lifestyle characteristics. Anthropometric measurements including height, weight and waist circumference were measured. Body mass index (kg/m^2^) was calculated, and lean body mass and fat percentage were estimated with bioelectrical impedance using the InBody 3.0 eight polar tactile electrode system (Biospace Co., Ltd., Seoul, Korea). Physical activity was assessed using a validated exercise questionnaire; the Kuopio Ischaemic Heart Disease Risk Factor Study (KIHD) 12-month leisure time physical activity (LTPA) history [30]. The questionnaire assigned a metabolic equivalent of task (MET) value for each specific activity and intensity. Physical activity is presented as MET-hours per day based on a 12-month history.

### Statistical analysis

Means and standard deviations or medians and interquartile ranges were calculated for continuous variables, whereas categorical variables are presented as frequencies and proportions. The associations between the characteristics of the cohort members and physical performance were assessed using linear regression analyses. Multiple linear regression models were used to assess the association between glucose regulation and physical performance. The results are presented pooled by sex, as none of the interactions for sex and the glucose regulation variables on the Senior Fitness Test were statistically significant (all *p* values > 0.52). In model 1, we adjusted for sex and age. Next, we added adult socioeconomic status, smoking status, alcohol consumption and physical activity to model 2. In model 3, we further adjusted for body fat percentage. All tests were performed two-tailed and the level of significance was set at *p* < 0.05. Statistical analyses were carried out using IBM SPSS Statistics Version 24 (IBM Corp., Armonk, NY, USA).

## Results

A total of 475 men and 603 women were included in the analysis (Table 1). The mean age at the baseline clinical examination was 61.2 (SD 2.6) years for men and 61.3 (SD 2.9) years for women. The prevalence of previously known diabetes was 6.7% for men and 3.0% for women. At the OGTT, 9.7% of the men and 5.1% of the women were diagnosed with new diabetes. Prediabetes had a higher prevalence, with 30.1% of the men and 29.2% of the women diagnosed with either IGT or IFG. The mean follow-up time between the OGTT and the SFT was 9.7 (SD 0.9) years for the entire cohort. Men had a mean age of 70.8 (SD 2.6) years and women a mean age of 71.0 (SD 2.8) years when physical performance was assessed.

**Table 1:** Baseline characteristics for men and women in the Helsinki birth cohort study

Variable	Men (*n* = 475)		Women (*n* = 603)	
	Mean	SD	Mean	SD
Age at baseline (years)	61.2	2.6	61.3	2.9
Weight (kg)	85.5	12.2	72.4	12.7
Height (cm)	177.2	5.9	163.4	5.7
BMI (kg/m^2^)	27.2	3.5	27.1	4.6
Lean body mass (kg)	65.5	7.3	47.6	5.3
Body fat (percentage)	22.9	5.5	33.2	6.6
Fat mass (kg)	19.9	7.1	24.6	8.9
Waist circumference (cm)	99.6	10.3	89.3	11.7
Smoking status (*r*/*n*, %)				
Never smoked	134/474	28.3	344/603	57.0
Ex-smoker	228/474	48.1	154/603	25.5
Smoker	112/474	23.6	105/603	17.4
Alcohol consumption (*r*/*n*, %)				
Never used/ex-user	29/471	6.2	28/600	4.7
Less than 1 day a week	126/471	26.8	311/600	51.8
Weekly	316/471	67.1	261/600	43.5
Adult socioeconomic status (*r*/*n*, %)				
Labourer	186/475	39.2	123/603	20.4
Lower middle	133/475	28.0	363/603	60.2
Upper middle	112/475	23.6	69/603	11.4
Self-employed	44/475	9.3	48/603	8.0
Self-reported physical activity (*r*/*n*, %)				
Sedentary	35/473	7.4	81/602	13.5
1–2 times/week	227/473	48.0	264/602	43.9
At least 3 times/week	211/473	44.6	257/602	42.7
LTPA (MET-h/Day)^a^	5.2	3.0 to 9.0	4.9	2.8 to 8.2
Education level (*r*/*n*, %)				
Basic	116/475	24.4	230/603	38.1
Upper secondary	115/475	24.2	157/603	26.0
Lower tertiary	143/475	30.1	159/603	26.4
Upper tertiary	101/475	21.3	57/603	9.5
Glucose regulation (*r*/*n*, %)				
Normoglycaemic	254/475	53.5	378/603	62.7
IFG	45/475	9.5	29/603	4.8
IGT	98/475	20.6	147/603	24.4
Newly diagnosed diabetes	46/475	9.7	31/603	5.1
Previously known diabetes	32/475	6.7	18/603	3.0

^a^ *LTPA* leisure time physical activity, values are given as median and interquartile ranges. *MET* metabolic equivalent of task, *IFG* impaired fasting glucose, *IGT* impaired glucose tolerance, Newly Diagnosed Diabetes: Diabetes diagnosed with oral glucose tolerance test at clinical examination during 2001–2004, Previously Known Diabetes: Diabetes diagnosed before clinical examination in 2001–2004

The mean overall SFT score was 45.0 (SD 17.5) points for the entire cohort. Women had a 4.40 point (*p* < 0.001) higher SFT score compared to men (Table 2). Baseline lean body mass and body fat percentage showed a significant inverse association with the SFT score. Subjects with previously known diabetes at baseline had a 17.77 lower mean SFT score (*p* < 0.001) compared to subjects with normoglycaemia. Those with newly diagnosed diabetes (*p* < 0.001) and IGT (*p* < 0.001) also had a significantly lower SFT score compared to their normoglycaemic counterparts. There was no significant difference in SFT score between those with IFG and those with normoglycaemia (*p* = 0.26).

**Table 2:** Associations (univariate) between subject characteristics and SFT score

Variable	*N* = 1078		
	*b*	95% CI	*p*
Age at senior fitness test (years)	− 0.09	− 0.48 to 0.29	0.64
Sex			
Men	Ref	–	–
Women	4.40	2.23 to 6.49	< 0.001
Height (cm)	− 0.10	− 0.21 to 0.02	0.11
Lean body mass (kg)	− 0.27	− 0.36 to − 0.17	< 0.001
Body fat (%)	− 0.51	− 0.64 to − 0.38	< 0.001
Smoking status			
Never smoked	Ref	–	–
Ex-smoker	− 1.00	− 3.31 to 1.32	0.40
Smoker	− 9.18	− 11.93 to − 6.42	< 0.001
Alcohol consumption			
Never used/ex-user	Ref	–	–
Less than 1 day a week	4.68	− 0.13 to 9.50	0.06
Weekly	5.92	1.17 to 10.66	0.02
Adult socioeconomic status			
Labourer	Ref	–	–
Lower middle	5.68	3.22 to 8.14	< 0.001
Upper middle	7.52	4.34 to 10.70	< 0.001
Self-employed	5.90	1.88 to 9.93	0.004
LTPA (MET-h/day)	0.15	− 0.05 to 0.35	0.14
Glucose regulation			
Normoglycaemic	Ref	–	–
IFG	− 2.37	− 6.47 to 1.74	0.26
IGT	− 5.15	− 7.65 to − 2.65	< 0.001
Newly diagnosed diabetes	− 10.55	− 14.56 to − 6.54	< 0.001
Previously known diabetes	− 17.77	− 22.65 to − 12.90	< 0.001

*SFT* senior fitness test, *b* regression coefficient (unstandardised), *LTPA* leisure time physical activity, *MET* metabolic equivalent of task, *IFG* impaired fasting glucose, *IGT* impaired glucose tolerance

Table 3 shows the association between glucose regulation and physical performance 10 years later. There was no significant difference in physical performance between subjects with IFG and subjects with normoglycaemia. Those with previously known diabetes, newly diagnosed diabetes and IGT all had a lower SFT score compared to those with normoglycaemia. There was a graded increase in the inverse association between more severe impairment in glucose regulation and physical performance, as subjects with IGT showed the weakest association and subjects with previously known diabetes showed the strongest inverse association with the overall SFT score. After adjusting for sex, age, adult socioeconomic status, and lifestyle factors, the regression coefficients were only slightly attenuated. However, further adjustment for body fat percentage had an impact on these associations. In the fully adjusted model, the mean difference in SFT score between subjects with previously known diabetes and normoglycaemic subjects was − 11.56 points (95% CI − 16.15 to − 6.98, *p* < 0.001).

**Table 3:** Regression coefficients for the association between glucose regulation and SFT score

Glucose regulation^a^	Model 1			Model 2			Model 3		
	*b*	95% CI	*p*	*b*	95% CI	*p*	*b*	95% CI	*p*
IFG	− 1.69	− 5.80 to 2.42	0.42	− 2.03	− 6.04 to 1.98	0.32	0.47	− 3.47 to 4.40	0.82
IGT	− 5.13	− 7.63 to − 2.63	< 0.001	− 5.31	− 7.77 to − 2.86	< 0.001	− 2.56	− 4.96 to − 0.16	0.04
Newly diagnosed diabetes	− 9.86	− 13.87 to − 5.84	< 0.001	− 8.97	− 12.89 to − 5.05	< 0.001	− 5.49	− 9.26 to − 1.72	0.004
Previously known diabetes	− 16.95	− 21.84 to − 12.07	< 0.001	− 15.72	− 20.54 to − 10.91	< 0.001	− 11.56	− 16.15 to − 6.98	< 0.001

^a^Normoglycaemia serves as the reference. Model 1: Adjusted for sex and age. Model 2: Further adjusted for adult socioeconomic status, smoking status, alcohol consumption and physical activity. Model 3: Further adjusted for body fat percentage. *SFT* senior fitness test, *b* regression coefficient (unstandardised), *IFG* impaired fasting glucose, *IGT* impaired glucose tolerance

The association between glucose regulation and the age standardised scores for the individual SFT tasks are presented in Table 4. In the fully adjusted model, subjects with previously known diabetes performed poorer in all tasks except the chair sit-and-reach test compared to those with normoglycaemia. The greatest difference between subjects with previously known diabetes and subjects with normoglycaemia was in the 6-min walk test (mean difference − 3.38, 95% CI − 4.76 to − 2.00, *p* < 0.001). Subjects with newly diagnosed diabetes performed significantly poorer in both the arm curl test and the back scratch test. Subjects with IGT performed poorer in the arm curl test, but there was no difference in the rest of the individual SFT tasks compared to subjects with normoglycaemia.

**Table 4:** Regression coefficients for the association between glucose regulation and individual SFT test components^a^

Glucose regulation^b^	Chair stand			Arm curl			Back scratch			Chair sit-and-reach			6-min walk		
	*b*	95% CI	*p*	*b*	95% CI	*p*	*b*	95% CI	*p*	*b*	95% CI	*p*	*b*	95% CI	*p*
IFG	0.32	− 0.57 to 1.21	0.48	− 0.37	− 1.52 to 0.79	0.53	− 0.33	− 1.75 to 1.08	0.65	− 0.14	− 1.60 to 1.32	0.85	1.08	− 0.10 to 2.25	0.07
IGT	− 0.29	− 0.84 to 0.25	0.29	− 0.71	− 1.43 to − 0.00	0.049	− 0.36	− 1.23 to 0.52	0.42	− 0.72	− 1.61 to 0.18	0.12	− 0.50	− 1.22 to 0.22	0.17
Newly diagnosed diabetes	− 0.73	− 1.58 to 0.12	0.09	− 1.48	− 2.60 to − 0.37	0.01	− 1.72	− 3.10 to − 0.35	0.01	− 0.51	− 1.92 to 0.90	0.48	− 1.00	− 2.14 to 0.14	0.09
Previously known diabetes	− 1.95	− 2.98 to − 0.92	< 0.001	− 2.25	− 3.61 to − 0.90	0.001	− 2.85	− 4.58 to − 1.12	0.001	− 1.14	− 2.84 to 0.57	0.19	− 3.38	− 4.76 to − 2.00	< 0.001

^a^Adjusted for sex, age, adult socioeconomic status, smoking status, alcohol consumption, physical activity and body fat percentage

^b^Normoglycaemia serves as the reference. *SFT* senior fitness test, *b* regression coefficient (unstandardised), *IFG* impaired fasting glucose, *IGT* impaired glucose tolerance

## Discussion

In this study of community dwelling older people, we found that impaired glucose regulation was strongly associated with decreased physical performance nearly 10 years later. We showed that physical performance was poorer not only among individuals with diabetes, but also among those with IGT, even after controlling for confounding factors. There was a gradual decline in physical performance when transitioning to more severe disturbances in glucose regulation, with subjects previously diagnosed with diabetes having the poorest physical performance. To the best of our knowledge, we are the first to report this kind of relationship between glucose regulation and physical performance in a longitudinal study setting.

We confirmed the results from previous studies, suggesting an association between diabetes and decreased physical performance. We found that after controlling for confounders, subjects with previously diagnosed diabetes had approximately 0.7 SD lower SFT score compared to subjects with normoglycaemia. In a meta-analysis by Wong and colleagues [12] including 26 studies, diabetes was shown to increase the risk of mobility disability, IADL disability and ADL disability. In a 7-year follow-up study of Mexican-Americans, diabetes was associated with roughly a 1.5–2 times greater risk of lower body disability compared to normoglycaemia [31]. Some data, although limited, are also available on the relationship between IGT and physical performance. In a cross-sectional study of 1391 individuals, both IGT and diabetes increased the risk of poor physical performance [32]. These results together with our findings suggest that physical performance is affected already at the stage of IGT.

Several mechanisms may explain the association between impaired glucose regulation and decreased physical performance. Insulin resistance has been shown to cause increased protein degradation and decreased protein synthesis in skeletal muscle. In the long term, this may cause loss of muscle mass, which further aggravates glucose regulation, as skeletal muscle is important for the uptake of glucose [33]. Inflammatory markers associated with insulin resistance and obesity, such as interleukin-6 and C-reactive protein (CRP), may also contribute to functional decline by causing negative changes in the regulation of skeletal muscle homeostasis [16]. In addition, physical inactivity, mitochondrial dysfunction, decreased skeletal muscle blood flow and hyperglycaemia itself may explain the excess disability among adults with abnormal glucose regulation [34].

In our analysis, adjusting for body fat percentage weakened the association between glucose regulation and physical performance. Body fat percentage and obesity are strongly associated with disturbances in glucose regulation and have been shown to be important predictors of poor physical performance [26, 35]. Excess body fat triggers low-grade systemic inflammation and may cause accumulation of both intermuscular and intramuscular fat deposits [36]. This has been shown to have a negative impact on muscle strength and increase the risk of mobility disability [34]. Additionally, ectopic fat also affects glucose metabolism and it is suggested that this could be one of the triggers of insulin resistance [33]. Functional decline and physical inactivity, on the other hand, further increases the risk of obesity, metabolic syndrome and other chronic diseases [37].

Compared to subjects with normoglycaemia, we found that people with previously known diabetes had, besides the lowest overall SFT score, also a poorer result on most SFT test components. This suggests that over time, many different elements of physical performance important in daily life are affected among those with diabetes, including strength, endurance and flexibility. Previously known diabetes had the strongest inverse association with the 6-min walk test, indicating that lower body function in particular is decreased among individuals with diabetes. Newly diagnosed diabetes, on the other hand, was inversely associated with only two out of five performance tests. This indicates that longer duration of diabetes is an important risk factor of decreased physical function [9, 13].

From a public health point of view, our results show optimistic possibilities for potentially slowing down functional decline among older people. By diagnosing impairments in glucose regulation at an early stage, lifestyle interventions and patient education might prevent further decline in glucose regulation [5], thereby promoting the maintenance of physical performance. This could ultimately lead to a reduction of health care costs and an increase in functional independence of older people [17]. Pharmacotherapy, such as metformin, reduces the risk of progression to diabetes among subjects with prediabetes, however, this treatment has been shown to be less effective compared to lifestyle interventions [38]. A topic for future research would be to investigate if metformin could slow down the decline in physical performance among individuals with IGT.

This study has several strengths. We studied a large population which comprised both men and women. The long follow-up time of approximately 10 years enabled us to evaluate the long-term association between glucose regulation and physical performance. We used the OGTT to assess glucose regulation and were thereby able to correctly classify subjects with a high sensitivity. Relying solely on fasting plasma glucose or HbA_1c_ when diagnosing diabetes has been shown to be less sensitive compared to using an OGTT, with nearly 50% of undiagnosed diabetes detected only with an OGTT [39]. Another strength is that we used the validated SFT to objectively measure physical performance among our subjects. Compared to testing single muscle groups, such as grip strength or gait speed, the SFT can be used to evaluate full-body physical performance, including strength, flexibility and endurance. Another popular test of physical performance, the Short Physical Performance Battery (SPPB), although effective in predicting disability, nursing home admittance and survival rates among older people, includes test components that have been shown to be either too easy or too difficult to perform [40].

This study also has some limitations. First, although we were able to adjust for several covariates, we did not adjust for complications associated with diabetes, including neuropathy, retinopathy and peripheral vascular disease. These factors have been shown to be associated with disability and functional impairment among older people [35, 41]. In addition, there may be other unmeasured variables affecting our results. Second, physical performance was not assessed at the baseline clinical examination, thus, we were not able to report potential changes in physical performance during the follow-up time. This prevented us from addressing causality. The fact that individuals with the longest duration of diabetes had the poorest physical performance suggests, however, that impaired glucose regulation causes a decline in physical performance over time. Third, we acknowledge the possibility of a selection bias, as only half of the subjects from the baseline clinical examination participated in the examination of physical performance. Fourth, we studied a homogenous population of Caucasians only living in a restricted area of Finland, and this should be taken into account when implementing our findings in other populations.

In summary, there is a strong association between disturbances in glucose regulation and poor physical performance among older people. Decline in physical function is known to begin already at an early stage of impaired glucose regulation, however, diabetes, and in particular, a long duration of diabetes further exacerbates this decline and affects several different aspects of physical performance among individuals with advanced age.
